# Relaxation of a dense ensemble of spins in diamond under a continuous microwave driving field

**DOI:** 10.1038/s41598-021-95722-z

**Published:** 2021-08-11

**Authors:** Jeson Chen, Oliver Y. Chen, Huan-Cheng Chang

**Affiliations:** 1 Institute of Atomic and Molecular Sciences, Academia Sinica, Taipei, 106 Taiwan; 2 Department of Electronic Engineering, Feng Chia University, Taichung, 40724 Taiwan

**Keywords:** Applied physics, Quantum physics

## Abstract

Decoherence of Rabi oscillation in a two-level quantum system consists of two components, a simple exponential decay and a damped oscillation. In dense-ensemble spin systems like negatively charged nitrogen-vacancy (NV^−^) centers in diamond, fast quantum state decoherence often obscures clear observation of the Rabi nutation. On the other hand, the simple exponential decay (or baseline decay) of the oscillation in such spin systems can be readily detected but has not been thoroughly explored in the past. This study investigates in depth the baseline decay of dense spin ensembles in diamond under continuously driving microwave (MW). It is found that the baseline decay times of NV^−^ spins decrease with the increasing MW field strength and the MW detuning dependence of the decay times shows a Lorentzian-like spectrum. The experimental findings are in good agreement with simulations based on the Bloch formalism for a simple two-level system in the low MW power region after taking into account the effect of inhomogeneous broadening. This combined investigation provides new insight into fundamental spin relaxation processes under continuous driving electromagnetic fields and paves ways to better understanding of this underexplored phenomena using single NV^−^ centers, which have shown promising applications in quantum computing and quantum metrology.

## Introduction

The negatively charged nitrogen-vacancy (NV^−^) center in diamond constitutes an appealing platform for the development of quantum computers^1,2^ and quantum metrology^3,4^. It is a highly unique solid-state spin system in that their spins are optically polarizable and can be detected and manipulated individually at room temperature^5,6^. Recent advancements in the research of single NV^−^ centers have led to significant progress in fundamental physics^7,8^ as well as novel applications in various fields ranging from quantum information^9,10^ to nanometrology^11–16^. By using single NV^−^ centers in ultrapure diamonds to minimize interactions with the intrinsic spin baths in hosting matrixes, high precision measurements based on the manipulation of single spins by microwave (MW) pulses have been achieved by a number of research groups worldwide^17,18^.

Although single NV^−^ centers are ideal candidates for quantum metrology applications, the measurements are technically demanding and time-consuming. Ensemble NV^−^ centers, in contrast, have the advantages of higher signal levels and larger detection volumes. These centers have been utilized to achieve large-scale sensing in both physics and biology^19–22^, and several schemes for ensemble quantum computation has also been proposed^23,24^. However, a critical challenge in this context is the significant reduction of the spin coherence time and, hence, a shorter duration for quantum operations when the defect density is high (e.g. 100 ppm N and 10 ppm NV)^25–27^. In addition, coupled charge-spin reactions could occur more readily upon photoexcitation of these dense-defect samples^28–30^. Therefore, to realize the practical use of NV^−^ ensembles, it is essential to investigate ensemble spin depolarization dynamics in detail.

Characterization of decays during Rabi oscillations (RO) is an important step in investigating the decoherence of quantum systems. Environmental information could be extracted from decoherence of RO under continuous wave (CW) driving fields due to the coupling between the quantum system and its surroundings^31^. In solid-state spin systems, the decoherence of RO gives clues to the randomly fluctuating spin bath as well as the spatially inhomogeneous electromagnetic field in such systems^18,32^. Understanding of the decoherence process of a quantum spin state is therefore of critical importance for applications of solid-state materials in quantum physics. In addition, investigating accumulated decoherence during ROs from successive control operations of a quantum bit (qubit) can potentially improve the outcome fidelity in quantum computing^33,34^.

**Figure 1:**
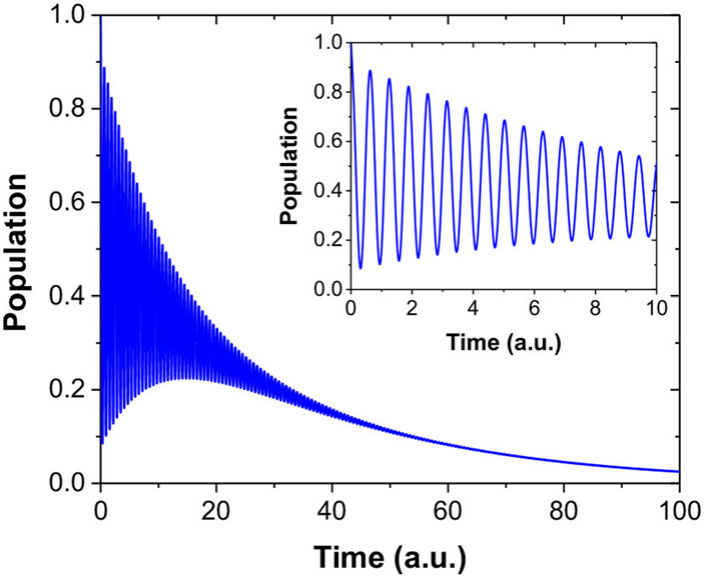
Pictorial representation of a damped Rabi oscillation in a two-level system. Inset: Enlarged view of the short time behavior of the relaxation.

The general solution of damped RO driven by a coherent electromagnetic field can be calculated from Bloch equations to give^35,36^:

1 $$f\left( t \right) = Ae^{ - \alpha t} + Be^{ - \beta t} \cos \left( {wt} \right) + \frac{C}{w}e^{ - \beta t} \sin \left( {wt} \right) + D,$$

where $f\left( t \right)$ is the time dependence of any components of a magnetic moment in three dimensions, $A$, $B$, $C$, $D$, $\alpha$, $\beta$, and $w$ are functions of Rabi frequency $\Omega_{R}$, detuning $\delta$, longitudinal relaxation time $T_{1}$, and transverse relaxation time $T_{2}$. According to this equation, the temporal dynamics of the quantum system under a CW driving field is composed of two terms: a baseline decay (i.e. a simple exponential decay function with the time constant $\alpha$) plus an oscillatory amplitude decay (i.e. an exponential function with the time constant $\beta$ multiplied by a sinusoidal or cosinusoidal function), as shown pictorially in Fig. 1. Presently, research in the field has been focused mainly on the oscillatory amplitude decay of single NV^−^ spins in diamond^18,37,38^, and little attention has been paid to the baseline decay in the Rabi damping process. Particularly, in-depth investigations of the extensively damped ROs of NV^−^ ensembles are still lacking.

**Figure 2:**
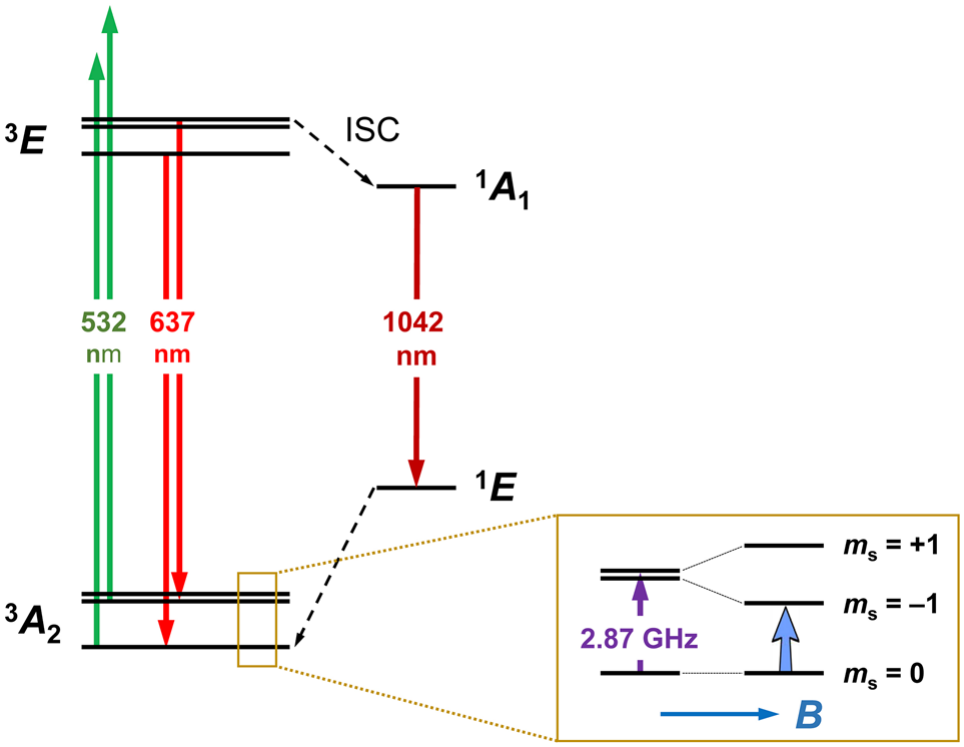
Energy level diagram of the NV^−^ center in diamond. *ISC* intersystem crossing.

Figure 2 presents the energy levels of NV^−^ centers in the presence of an external magnetic field. The zero-phonon line between the orbital ground and excited states of the center is located at 637 nm. In the absence of an external magnetic field, there is a transition (zero field splitting) at a frequency of 2.87 GHz between the $\left| {m_{s} = 0} \right\rangle$ and $\left| {m_{s} = \pm 1} \right\rangle$ spin sublevels of the orbital ground state. The spin degeneracy of this triplet system is readily lifted by an applied static magnetic field $B$, yielding a Zeeman energy splitting of $2\gamma B$ between $\left| {m_{s} = - 1} \right\rangle$ and $\left| {m_{s} = + 1} \right\rangle$ sublevels, where $\gamma$ is the gyromagnetic ratio of the electron spin. When excited by green–yellow light, the laser illumination can continuously polarize the electron spins to the $\left| {m_{s} = 0} \right\rangle$ sublevel^39,40^, which enables optical initialization of the spin state. Additionally, the far-red fluorescence light emitted from the center differs by ~ 30% between $\left| {m_{s} = 0} \right\rangle$ and $\left| {m_{s} = \pm 1} \right\rangle$ spin sublevels, due to intersystem crossing of $\left| {m_{s} = \pm 1} \right\rangle$ sublevels to a largely non-radiative channel with a lifetime of ~ 300 ns^39,41–43^. Both the optical spin initialization and optical readout of spin states form the basis for optically detected magnetic resonance (ODMR) of NV^−^ centers in diamond.

This work is aimed to identify and also measure experimentally the baseline decays of NV^−^ spin ensembles in diamond under CW MW driving fields. Specifically, we measured the MW-driven baseline decays of the dense NV^−^ ensembles by recording the decays of fluorescence signals under CW MW irradiation at various powers and frequencies resonant between two of the three spin sublevels (Fig. 2). The sample chosen for this study is a type-Ib diamond microcrystal containing ~ 150 ppm of substitutional nitrogen atoms and ~ 10 ppm of NV centers, produced by electron irradiation and subsequent annealing^44,45^. In addition, to comprehend the experimental observations, we performed numerical simulations for the baseline decays of an ensemble of spins during RO based on the two-level Bloch equations. Results of this combined study are expected to shed light on this underexplored phenomenon and supply useful information on the potential applications of dense spin ensembles in quantum metrology, quantum sensing, and related research areas.

## Results and discussion

### Experiment

**Figure 3:**
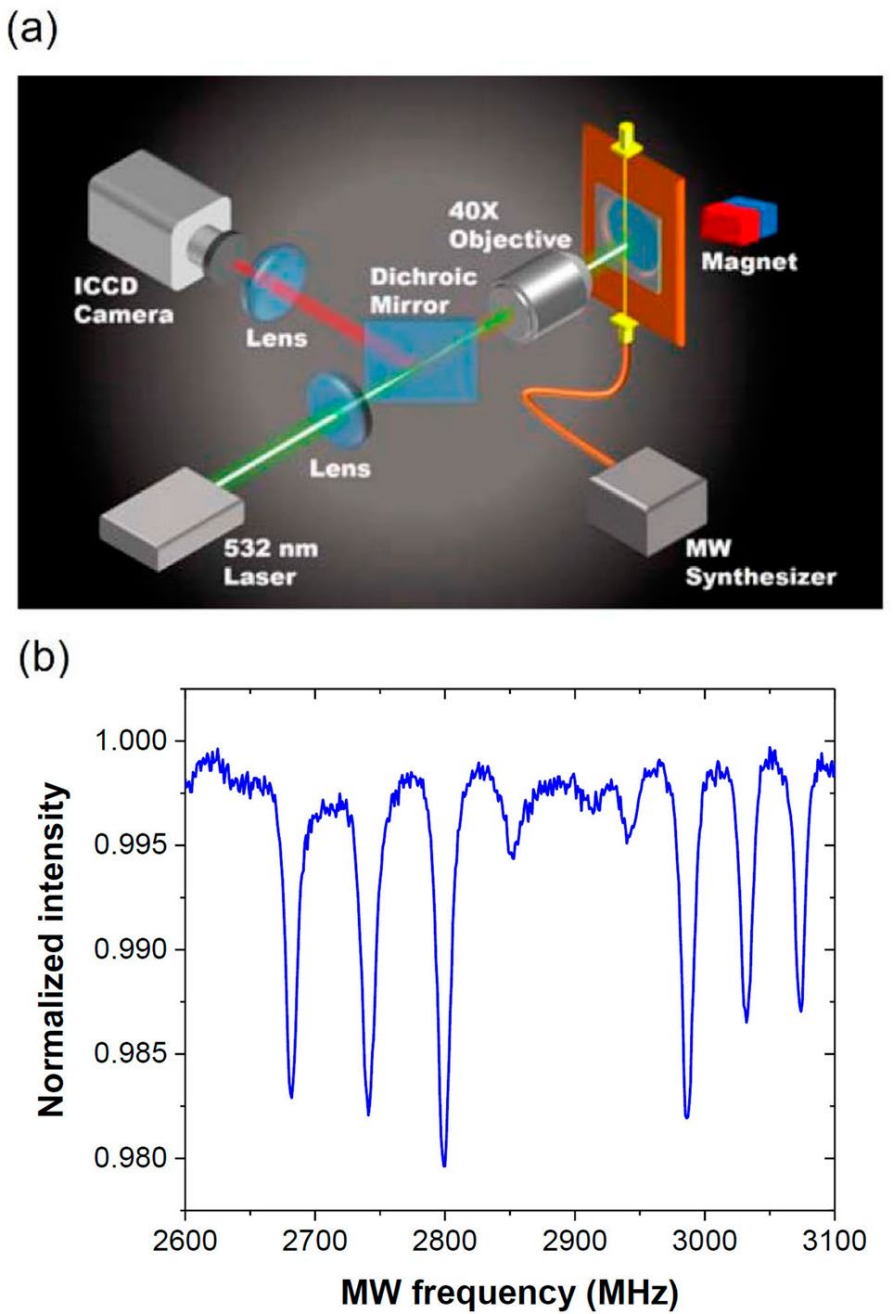
(**a**) Schematic diagram of the experimental setup. The sample consists of a diamond microcrystal containing a dense ensemble of NV^−^ centers. (**b**) Typical normalized ODMR spectrum of the NV^−^ centers in FMD, acquired in the presence of an external magnetic field of $B_{\parallel }$ = 6.6 mT.

All measurement was performed with a home-built wide-field fluorescence imaging setup. The setup consisted of a 532-nm continuous-wave laser for optical pumping and probing, and an intensified charge-coupled device (ICCD) camera for photon detection and fluorescence imaging (Fig. 3a). The tested sample consisted of a fluorescent microdiamond (FMD) crystal of ~ 100 μm in diameter and ~ 10 ppm in NV density glued to a glass coverslip. A static magnetic field was applied onto the sample to lift the spin degeneracy. Figure 3b shows a typical optically detected magnetic resonance (ODMR) spectrum of this diamond sample exposed to a magnetic field of $B_{\parallel } \approx$ 6.6 mT. The spectrum was acquired by summing together the data of all pixels in the ICCD images to enhance the signal-to-noise ratios. From the continuous-wave ODMR spectra, we obtained the spin resonance frequencies in the region of interest and used them as the references for ensuing MW-driven spin relaxation measurements.

**Figure 4:**
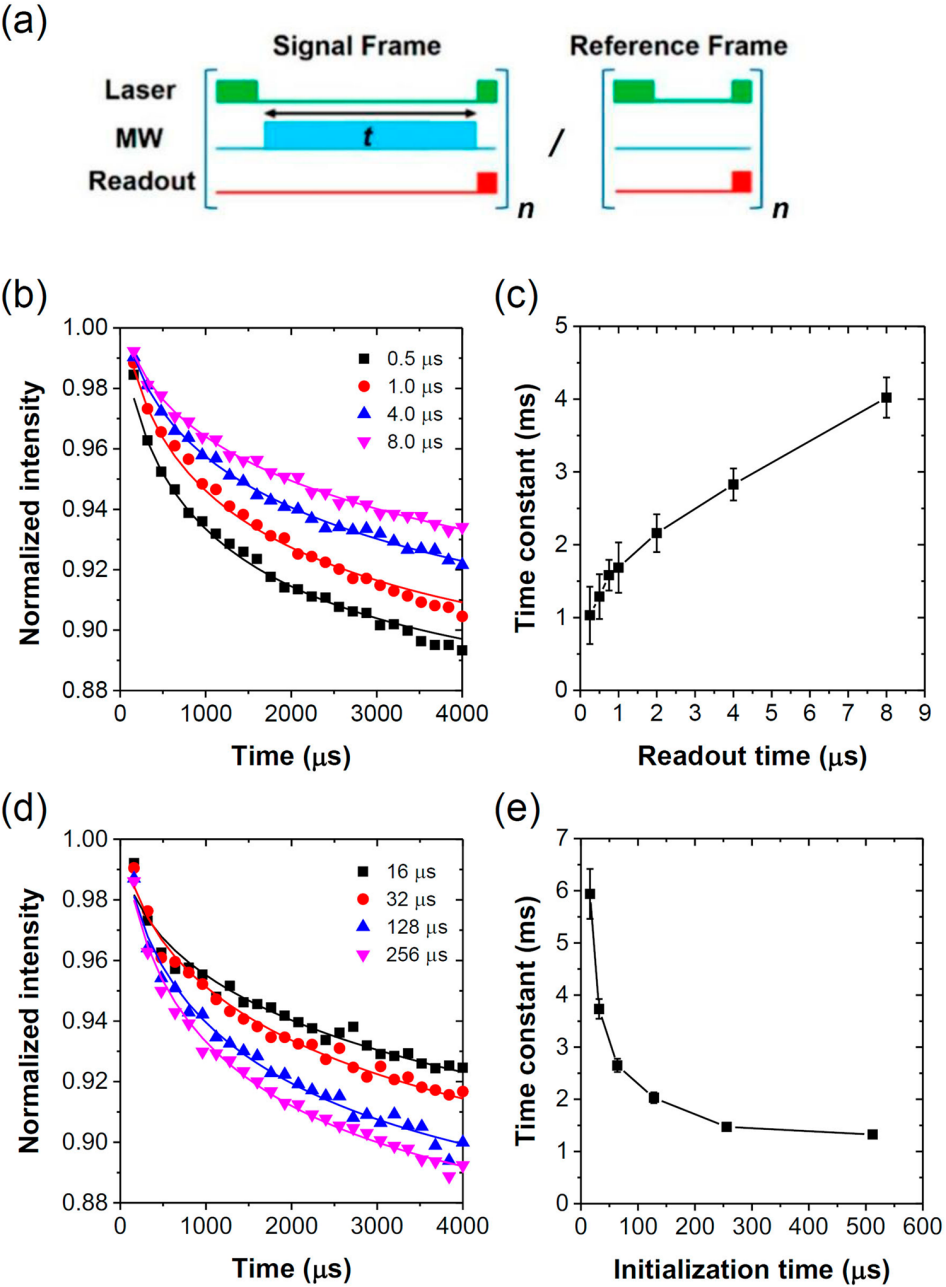
(**a**) Pulse sequences for relaxation measurements. A signal frame with variable delay time $t$ was followed by a fixed delay reference frame. MW irradiation was applied during the variable delay time $t$ in between laser pulses for Rabi/driven relaxation experiments, while MW was absent during the variable delay time $t$ in between laser pulses for MW-free depolarization experiments. Normalization of signals was achieved by dividing a signal frame to its follow-up reference frame. (**b**) Depolarization measurements made with different durations of readout pulses. The normalized signals were fitted with a stretched-exponential function to obtain the decay time constant $T_{off}$. (**c**) Measured depolarization decay time constant $T_{off}$ as a function of readout time. The length of the initialization pulses in (**b**) and (**c**) is 300 μs. (**d**) Depolarization measurements made with different durations of initialization pulses. The normalized signals were fitted with a stretched-exponential function to obtain the decay time constant $T_{off}$. (**e**) Measured depolarization decay time constant $T_{off}$ as a function of initialization time. The length of the readout pulses in (**d**) and (**e**) is 0.5 μs.

In studying the spin relaxation dynamics, it is important to ensure that the pulse durations of both laser initialization and readout time are optimized for the depolarization measurements. To address this issue, we first characterized MW-free depolarization with different durations of initialization and readout pulses (cf. Fig. 4a for the pulse sequences). The photoluminescence signals were collected with an ICCD camera synchronized with our applied pulse sequences, which consisted of a laser initialization pulse, followed by a variable duration $t$ and then optical detection with a readout pulse. There was a 2-μs decay between the initialization light pulse and the variable duration time $t$ as well as a 2-μs decay between the variable duration time $t$ and the readout light pulse. The entire sequence was repeated by 90 times within the exposure time of the camera as the signal frame. After a signal frame, we alternated a reference frame having a fixed delay of 2 μs between initialization and readout pulses and repeated the detection by 90 times. The signal frame and reference frame was alternating in sequence to remove low frequency noise and drifting during laser illumination. We then divided signals obtained from the signal frame by its reference frame signals to correct (or normalize) laser intensity fluctuations. By introducing MW during the variable duration time $t$, the same pulse sequence is also used for MW-driven relaxation experiments which will be described in more detail later. Figure 4b shows the photoluminescence decay data obtained with different durations of the readout pulses but with the same initialization pulse of 300 μs in the absence of MW. Instead of a simple exponential function, each spin depolarization trace could only be well fitted with a stretched-exponential equation of the form

2 $$I\left( t \right) = I_{0} \exp \left[ { - \left( {\frac{t}{{T_{off} }}} \right)^{\beta } } \right] + I_{eq} ,$$

where $I_{0}$ is the amplitude of the exponential decay, $T_{off}$ is the decay time constant when the MW is off, $\beta$ is a sample-dependent index, and $I_{eq}$ is the photoluminescence baseline. With iterative least squares estimation method, the fitted regression function with minimum errors gave an estimated $\beta$ ~ 0.5, which allowed good fits of all curves. The result is consistent with a previous report for stretched-exponential decays in NV^−^ spin ensembles^26,27^ as well as the macroscopic theory of Choi et al.^46^ for ensemble spin relaxation.

In Fig. 4c, we also display the fitted spin depolarization times, plotted against the durations of the readout pulses with the same initialization pulses of 300 μs in length. Since the lower metastable singlet state of the NV^−^ center has a lifetime of ~ 0.3 μs^43^, the minimum optical readout duration was chosen to be at least 0.5 μs to make sure the electrons relaxed to its orbital ground state. The measured decay time constants were observed to increase monotonically with the increasing readout pulse durations. In the long readout time extremum, we could infer that the electron spins received abundant laser photons and were repolarized close to their initial states. As a result, the long readout signals were barely decayed with respect to the initial signals and the decay time constants also approached infinity in this long readout time extremum. Figure 4d and e show the photoluminescence decay data obtained with different durations of initialization pulses implemented with the same readout pulses of 0.5 μs in length. The degree of spin depolarization monotonically increased with a longer initialization pulse duration and it leveled off when the initialization pulse duration was more than 300 μs at our laser power settings. Unlike the spin polarization time of 1–5 μs for single NV^−^ centers, it took about 300 μs to reach saturation of the spin polarization in our ensemble sample, comparable to 200–500 μs found by other studies for NV^−^ centers with a similar density^29,30,46^. The observed initialization-dependent depolarization could not be explained solely by simple spin dynamics, and we attributed the phenomena to the coupled spin-charge dynamics for diamonds with a dense ensemble of NV^−^ centers^29,30,46^. The spin polarization/depolarization dynamics in this kind of spin ensemble is intricate due to the interplay of spin polarization and photoionization during the laser illumination as well as recharging of the centers by reacting with nearby nitrogen atoms in the dark. Nonetheless, the results presented in Fig. 4 justify the use of an initialization pulse duration of 300 μs and a readout pulse duration of 0.5 μs in ensuring MW-driven relaxation experiments.

In addition to the pulse durations, we have also investigated the interference from the co-excitation of both unpolarized NV^−^ centers and NV^0^ centers in the FMD by the green laser^28–30^. In particular, the charge recombination process between the photoionized NV^−^ centers and the substitutional nitrogen atoms, i.e. NV^0^ + N^0^ → NV^−^ + N^+^, in our sample was carefully examined. We applied the pulse sequences illustrated in Fig. 4a with a step size of 1 μs to study the charge dynamics of the NV centers after photo-induced spin polarization with different laser powers (7 mW and 70 mW). A rising component associated with the charge recombination was detected at the beginning of the photoluminescence time trace in both cases (Fig. S1 in Supplementary Information). The time constant of this component was less than 2 μs, which was substantially smaller than the step size of 160 μs used in baseline decay measurements. The effect is thus ignored.

**Figure 5:**
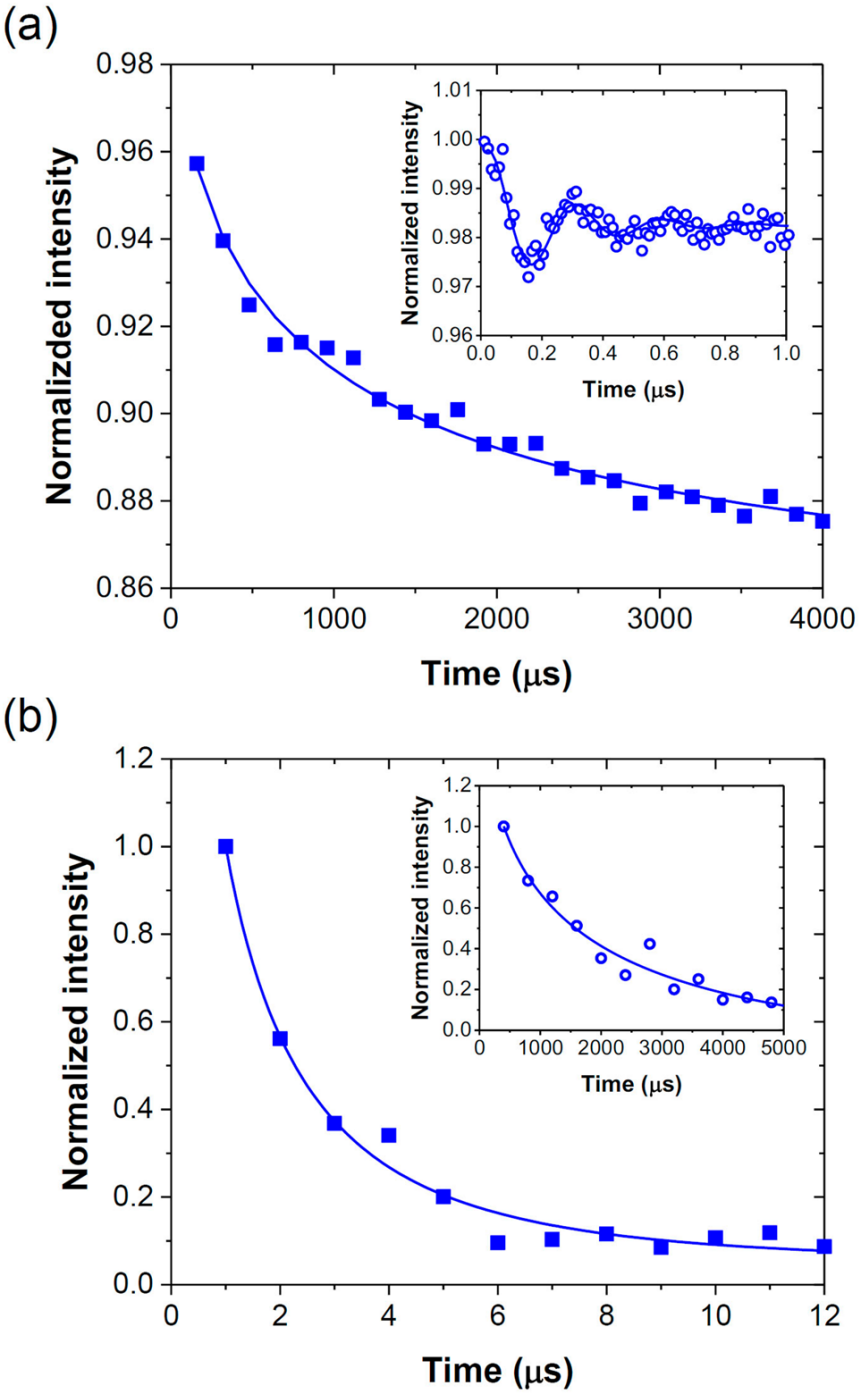
(**a**) Measurement for the MW-driven relaxation time at 2682 MHz. Inset: Zoom-in relaxation data of the same MW in a 1-μs time window for the measurement of Rabi frequency. (**b**) Measurement for the spin–spin relaxation time with $T_{2}$ = 1.7 μs. The times labeled in the x-axes are the durations between pairs of MW π/2 pulses ^43^. Inset: Measurement for the spin–lattice relaxation time with $T_{1}$ = 1491 μs. The times labeled in the *x*-axes are the durations between MW π and optical readout pulses^27^. Solid curves are best fits of the experimental data.

Prior to measuring the MW-driven damping time, we first detected the Rabi nutation at high MW powers. Specifically, we applied MW radiation with a variable duration of $t$ and a fixed delay of 2 μs between MW and laser pulses to ensure that the spins were completely relaxed from the metastable singlet states to the ground triplet states after photo-induced spin polarization. With the on-resonance MW excitation at 2682 MHz, we observed the Rabi nutation, whose time trace could be well characterized by a damped cosine wave with a frequency of $\Omega_{R} {/}2\pi$ = 3.44 MHz (inset of Fig. 5a). The amplitude of the Rabi oscillation decayed within 1 μs, most likely due to the inhomogeneity in the MW field strength across the sample as well as the inhomogeneous broadening of the spin transitions from bath, which jointly dephase the oscillation. Also, no beating between transitions of the ^14^N hyperfine structures in NV^−^ was observable presumably due to the detuning effect in combination with ensemble averaging. More importantly, however, we did observe a slower decay in the spin depolarization process, occurring at the millisecond time scale (Fig. 5a). Clearly, the MW-driven spin relaxation, i.e. damping of RO, consists of two components: a microsecond-scale “fast” decay component related to the oscillating amplitude decay and a millisecond-scale “slow” decay component related to the baseline decay, a picture consistent with Eq. (1).

Next, we measured the spin–spin relaxation time with the Hahn echo technique following the standard procedures described in the literature^25^ and obtained a bath-decoupled $T_{2} { }$ = 1.70 ± 0.13 μs (Fig. 5b). The value is about 3 orders of magnitude smaller than $T_{1}$ = 1491 ± 257 μs (inset of Fig. 5b), which is a typical spin–lattice relaxation time reported for NV^−^ centers in type-Ib diamonds^26,27^. These two time constants serve as the most important references for subsequent analysis of the Rabi baseline relaxation times, $T_{b}$. Following the notation of Jensen et al.^47^, we expressed the applied MW powers in terms of Rabi frequencies ($\Omega_{R} {/}2\pi$) in the ensuing discussion by referring to the result presented in the inset of Fig. 5a. Furthermore, to reduce the photoionization and charge recombination effects on the measurements of $T_{b}$^29,30^, we kept the laser powers low at ~ 1 kW/cm^2^ throughout this study. No shifting of the peaks in the ODMR spectrum due to heating near the MW wire was observed when $\Omega_{R} {/}2\pi$ < 1 MHz.

In extracting $T_{b}$ from experimental data, two complications appeared in the fitting of the measured time traces. First, the laser was illuminated on both MW-resonant and MW-non-resonant NV^−^ centers coexisting in the FMD crystal and therefore non-MW-driven NV^−^ centers could also emit photons and contribute to the observed relaxation. We characterized these MW-off-resonance photoluminescence decays with the time constant $T_{off}$ fitted from Eq. (2) for the MW-free depolarization. Second, the contribution from the transition $\left| {m_{s} = 0} \right\rangle$ → $\left| {m_{s} = - 1} \right\rangle$ or $\left| {m_{s} = 0} \right\rangle$ → $\left| {m_{s} = + 1} \right\rangle$ to the total relaxation was not a constant but varied with the MW power. For the on-resonance excitation at 2682 MHz, we assume that the ratio of the contribution from $\left| {m_{s} = 0} \right\rangle$ → $\left| {m_{s} = - 1} \right\rangle$ to $\left| {m_{s} = 0} \right\rangle$ → $\left| {m_{s} = + 1} \right\rangle$ is inversely proportional to the ratio of their respective lifetimes, i.e. $T_{b} / T_{off}$. We could therefore write the photoluminescence intensity decay in a double-stretched exponential form

3 $$I\left( t \right) = \frac{{I_{0} }}{4}\left[ {\left( {\frac{{T_{off} }}{{T_{off} + T_{b} }}} \right) \cdot \exp \left( { - \sqrt {\frac{t}{{T_{b} }}} } \right) + \left( {3 + \frac{{T_{b} }}{{T_{off} + T_{b} }}} \right) \cdot \exp \left( { - \sqrt {\frac{t}{{T_{off} }}} } \right)} \right] + I_{eq} ,$$

as derived in Supplementary Information. The fitting parameter $I_{eq}$ gives the equilibrium intensity at the long time extremum, and setting the lower threshold of the decay in the MW-driven damping experiment. Note that Eq. (3) is a modified scenario to Eq. (1) where non-MW-driven optical defects in diamond also contribute to the signals. The MW-driven component has to be extracted first from Eq. (3) and then analyzed by the damped RO described by Eq. (1).

**Figure 6:**
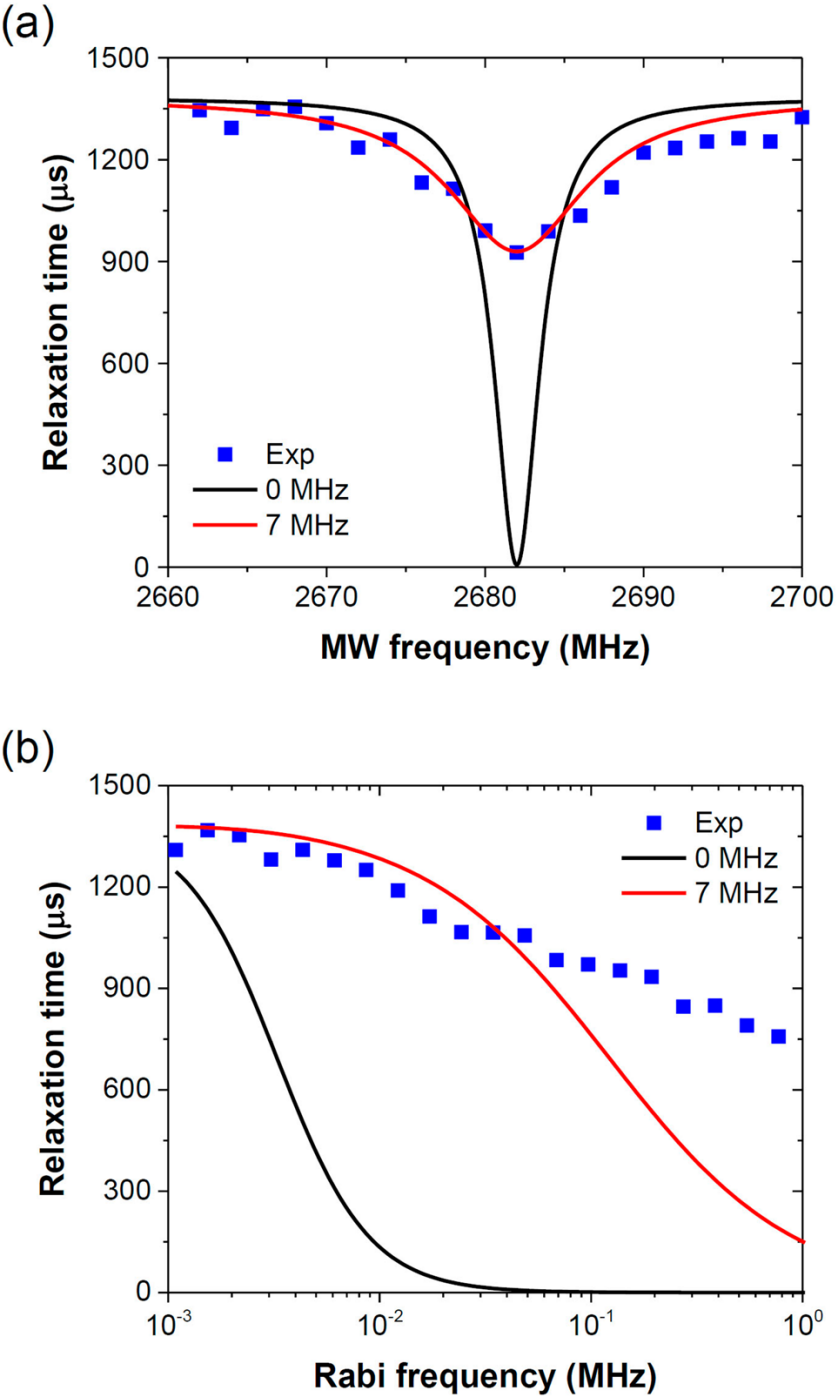
(**a**) Measured and simulated baseline decay time constants as a function of MW frequency at the magnetic field strength of $\Omega_{R} {/}2\pi$ = 0.061 MHz. (**b**) Measured and simulated baseline decay time constants as a function of MW magnetic field strength ($\Omega_{R} {/}2\pi$) at the MW frequency of 2682 MHz. Black and red curves in (**a**) and (**b**) are calculated from Eq. (16) with $\Delta \omega_{fs} {/}2\pi$ = 0 and 7 MHz.

Figure 6a shows the measured Rabi baseline relaxation times, extracted by fitting the experimental data to Eq. (3), as a function of the MW frequency over $\omega {/}2\pi$ = 2660–2700 MHz at the MW power of $\Omega_{R} {/}2\pi$ = 0.061 MHz. A change of $T_{b}$ by 32% was detected upon near-resonance excitation (i.e. $\delta \cong 0$). This change is substantially larger than the corresponding contrast of 0.3% in continuous-wave ODMR performed by using the same experimental setup and the same MW power. The frequency spectra of Rabi baseline relaxation time were repeated over different FMD crystals, orientations, and magnetic field strength, and the spectra all showed the same dip at the ODMR resonance frequencies. In addition to the frequency dependence measurements in the low MW power region ($\Omega_{R} {/}2\pi$ = 0.061 MHz), we also explored how the decay times might vary with the magnetic field strength upon on-resonance excitation (i.e. $\delta$ = 0). As shown in Fig. 6b, a decrease of the decay time with increasing $\Omega_{R} {/}2\pi$ was found and the extent of the decrease could be up to 50% at the high MW power region with $\Omega_{R} {/}2\pi$ > 1 MHz.

### Simulation

To understand the MW frequency and power dependence of the baseline decay accompanied with RO, we used a density matrix for a simple two-level system to model the spin states for a single NV^−^ center and describe how the spin state of the NV^−^ center decoheres from a pure state to a mixed state. The Bloch equations to model the dynamics of the spin are^47^

4 $$\frac{d}{dt}\left( {\begin{array}{*{20}c} {\rho 00\left( t \right)} \\ {\rho 01\left( t \right)} \\ {\rho 10\left( t \right)} \\ {\rho 11\left( t \right)} \\ \end{array} } \right) = \left( {\begin{array}{*{20}c} { - \varGamma_{1} } & {i\Omega } & { - i\Omega } & {\varGamma_{1} } \\ {i\Omega } & { - \varGamma_{2} + i\delta } & 0 & { - i\Omega } \\ { - i\Omega } & 0 & { - \varGamma_{2} - i\delta } & {i\Omega } \\ {\varGamma_{1} } & { - i\Omega } & {i\Omega } & { - \varGamma_{1} } \\ \end{array} } \right)\left( {\begin{array}{*{20}c} {\rho 00\left( t \right)} \\ {\rho 01\left( t \right)} \\ {\rho 10\left( t \right)} \\ {\rho 11\left( t \right)} \\ \end{array} } \right),$$

where $\rho 00\left( t \right)$ and $\rho 11\left( t \right)$ are the populations of $\left| {m_{s} = 0} \right\rangle$ and $\left| {m_{s} = - 1} \right\rangle$ (or $\left| {m_{s} = + 1} \right\rangle$) sublevels of the NV^−^ center, respectively, $\rho 01\left( t \right)$ and $\rho 10\left( t \right)$ are the coherences, and $\varGamma_{1}$ and $\varGamma_{2}$ are the spin–lattice and spin–spin relaxation rates defined as $\varGamma_{1} = 1/2T_{1}$ and $\varGamma_{2} = 1/T_{2}$, where the additional factor of 1/2 for $T_{1}$ is introduced so as to simplify Eq. (4). $\Omega = \Omega_{R} {/}2$ is half of the Rabi frequency of the driven electron spin, and $\delta = \omega - \left( {D + \gamma_{e} B_{\parallel } } \right)$ is the MW detuning with respect to the spin transition frequency $\left( {D + \gamma_{e} B_{\parallel } } \right)$, in which $B_{ \bot }$ is the magnitude of the linearly polarized magnetic field from MW (or the orthogonal component with respect to the NV axis), $\omega$ is the angular frequency (in rad/s) of the driving MW, and $B_{\parallel }$ is the parallel component of the applied static magnetic field along the NV axis. The solution of the $\left| {m_{s} = 0} \right\rangle$ population to Eq. (4) is of the form

5 $$\rho 00\left( t \right) = C_{1} e^{{\lambda_{1} t}} + C_{2} e^{{\lambda_{2} t}} + C_{3} e^{{\lambda_{3} t}} + C_{4} e^{{\lambda_{4} t}} ,$$

with $\lambda_{1} , \lambda_{2} , \lambda_{3} ,\lambda_{4}$ are complex eigenvalues of the matrix. To obtain these four eigenvalues, we need only to solve a third-degree polynomial equation given by

6 $$\lambda^{3} + 2\left( {\varGamma_{1} + \varGamma_{2} } \right)\lambda^{2} + \left( {4\Omega^{2} + 4\varGamma_{1} \varGamma_{2} + \varGamma_{2}^{2} + \delta^{2} } \right)\lambda + 2\left( {2\Omega^{2} \varGamma_{2} + \varGamma_{1} \varGamma_{2}^{2} + \varGamma_{1} \delta^{2} } \right) = 0,$$

as one of the eigenvalues is 0. The eigenvalue with $\lambda = 0$ is a trivial solution that corresponding to a stationary state, which is not of interest here. The remaining tasks are to solve these complex eigenvalues $\lambda$ in terms of Rabi frequency $\Omega$ and detuning $\delta$, which corresponding to the decay rates. Although there is no upper limit for achievable Rabi frequency here for a circularly polarized MW source, care must be taken in the high Rabi frequency regions from a linear polarized MW source, where the simple rotating-wave approximation no longer holds and results in non-linear responses to the driven electromagnetic waves^34,48^.

In samples of dense spins, the individual spin encounters random detuning at any given MW frequency from a quasi-static spin bath noise^49^. For dense ensembles of NV^−^ centers in bulk diamonds, the inhomogeneously broadened spin resonance linewidths can be as large as 3 MHz^47^. To treat the general cases where the detuning is nonzero (i.e. $\delta \ne 0$), we begin the analysis by numerically solving the polynomial equation given in Eq. (6). In these cases, the eigenvalues of the matrix in Eq. (4) generally consist of 1 real number and 2 complex conjugates, and the solution in form of Eq. (5) can be written as

7 $$\rho 00\left( t \right) = C_{1} *e^{{\lambda_{b} t}} + C_{2} *e^{{\lambda_{r} t}} \cos \left( {\lambda_{i} t + \theta } \right) + C_{3} ,$$

where $\lambda_{b}$ is the real eigenvalue, $\lambda_{r}$ are $\lambda_{i}$ are the real and imaginary parts of these 2 complex conjugate eigenvalues, respectively, and $\theta$ is the phase angle. As noted, the decay of the Rabi oscillatory amplitude is governed by $\lambda_{r}$ and the angular frequency of the Rabi nutation is described by $\lambda_{i}$. By comparing with a cubic root equation to Eq. (6):

8 $$\left( {\lambda - \lambda_{1} } \right)\left( {\lambda - \lambda_{2} } \right)\left( {\lambda - \lambda_{3} } \right) = 0,$$

we have

9 $$\lambda_{1} + \lambda_{2} + \lambda_{3} = \lambda_{b} + 2\lambda_{r} = - 2\left( {\varGamma_{1} + \varGamma_{2} } \right).$$

Furthermore, by factoring Eq. (6) into

10 $$4\Omega^{2} \left( {\lambda + \varGamma_{2} } \right) + \left( {\lambda + 2\varGamma_{1} } \right)\left( {\lambda^{2} + 2\varGamma_{2} \lambda + \varGamma_{2}^{2} + \delta^{2} } \right) = 0,$$

one can determine the upper bound, $\lim_{\Omega \to 0} \lambda_{b} = - 2\varGamma_{1}$, and the lower bound, $\lim_{\Omega \to \infty } \lambda_{b} = - \varGamma_{2}$, of the baseline decay rate. The analysis indicates that the value of $T_{b} \equiv - 1{/}\lambda_{b}$ is bounded by the spin’s intrinsic properties. When the driven MW power approaches zero (i.e. $\Omega \to 0$), $T_{b} \to T_{1}$ and the spin–lattice relaxation time $T_{1}$ sets the upper bound of $T_{b}$. When the driven MW power approaches infinity (i.e. $\Omega \to \infty$), $T_{b} \to T_{2}$ and the spin–spin relaxation time $T_{2}$ sets the lower bound of $T_{b}$. Therefore, we have a comparison of $T_{2} < T_{b} < T_{1}$. It can be seen that the oscillatory amplitude decay time, defined as $T_{r} \equiv - 1/\lambda_{r}$, could then be compared to $T_{1}$ and $T_{2}$ as $T_{2} < T_{r} < 2T_{1} T_{2} {/}\left( {T_{1} + T_{2} } \right)$ from Eq. (9). Given $\varGamma_{2} \gg \varGamma_{1}$ in typical spin systems, the oscillatory amplitude decay time $T_{r}$ is generally smaller than the baseline decay time $T_{b}$, and we could make a comparison of the relaxation time constants to be $T_{2} < T_{r} < 2T_{2}$.

**Figure 7:**
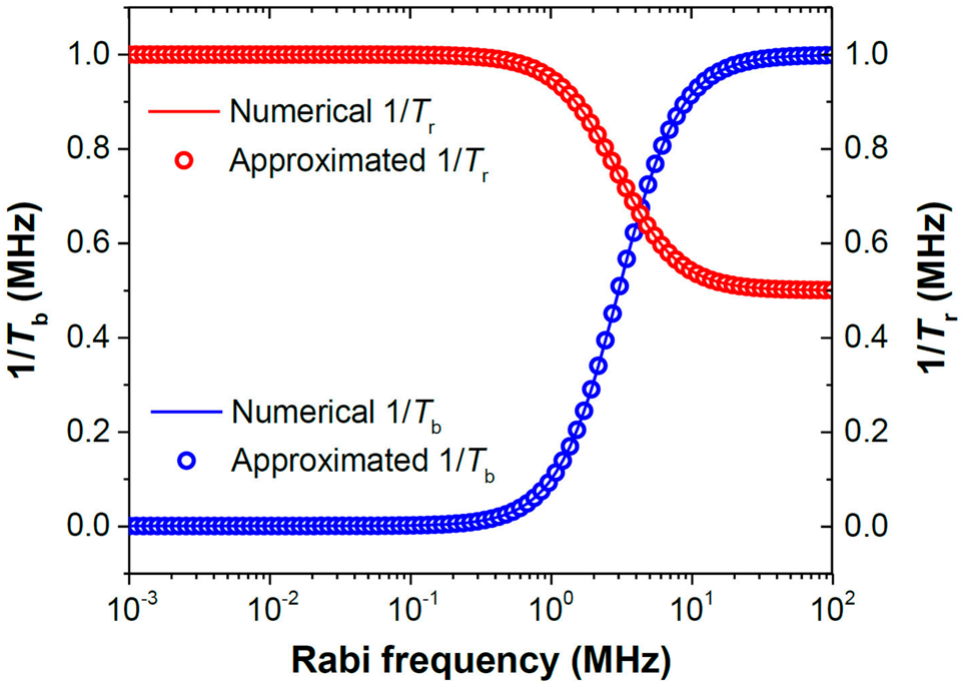
Single spin Rabi damping under the influence of external MW with $T_{1}$ = 1000 μs, $T_{2}$ = 1 μs, and $\delta {/}2\pi = 3\;{\text{MHz}}$. The left axis (blue) represents the baseline decay rate constant $1{/}T_{b}$ and the right axis (orange) represents the oscillatory amplitude decay rate constant $1{/}T_{r}$. Solid curves: numerical calculations; Open circles: calculations from Eqs. (11) and (12).

Taking $T_{1}$ = 1000 μs and $T_{2}$ = 1 μs as an example, we show in Fig. 7 the calculated results of $1{/}T_{b}$ and $1{/}T_{r}$ at $\delta {/}2\pi = 3$ MHz with the MW power expressed in terms of Rabi frequency ($\Omega_{R} {/}2\pi$). Starting with $1{/}T_{b} \left( \Omega \right) = 1{/}T_{1}$, the value of the baseline rate constant increases steadily as the MW power increases. On the other hand, the oscillatory amplitude decay rate $1{/}T_{r}$ shows an opposite trend to the driving MW power with respect to $1{/}T_{b}$, as predicted by Eq. (9). In Fig. 6b, we compare directly the measured and simulated baseline decay time constants $T_{b}$ as a function of MW magnetic field strength ($\Omega_{R} {/}2\pi$) at the MW frequency of 2682 MHz. Clearly, the numerical calculation significantly overestimates the power dependence, although a qualitative agreement in the trend of change between these two sets of data is reached.

For easier understanding of the frequency dependence of the baseline decay, it is preferable to provide an analytical form of $T_{b} \left( \Omega \right)$, even if it is an approximated one. By analyzing the numerical results calculated from the Bloch formalism in Eq. (6), we found empirically (cf. Fig. 7 and Fig. S2) that $T_{b} \left( \Omega \right)$ could be well described by the following equation,

11 $$T_{b} \left( {\Omega ,\delta } \right) = \frac{{T_{1} - T_{2} }}{{1 + \frac{{\Omega^{2} }}{Q}}} + T_{2} = \left( {T_{1} - T_{2} } \right)\left( {1 - \frac{{\Omega^{2} }}{{\Omega^{2} + Q}}} \right) + T_{2} ,$$

where $Q$ is a function of $\delta$. The equation can properly reproduce the two extremes discussed above for $T_{b}$. In addition, this form matches the observations that the dependence of $T_{b}$ on Rabi frequency in decibel unit is a logistic (or sigmodal) function. At $T_{1} \gg T_{2}$, a condition applicable for dense spin ensembles, the term $Q\left( \delta \right)$ can be simplified as

12 $$Q\left( \delta \right) = \frac{1}{{4T_{1} T_{2} }} + \frac{{T_{2} }}{{4T_{1} }}\delta^{2} ,$$

derived with an approximation detailed in Supplementary Information. Basically, $Q\left( \delta \right)$ is the sum of a constant plus a function proportional to $\delta^{2}$, which gives $T_{b}$ a Lorentzian-like profile with respect to detuning $\delta$. Furthermore, both the $\Omega$ and $\delta$ dependence of this analytical function $T_{b} \left( \Omega \right)$ is similar to the form observed for ODMR signals^47^. The full width at half maximum ($\Delta \omega_{b}$) of the peak in this relaxation time-detuning spectrum is

13 $$\Delta \omega_{b} = \frac{2}{{T_{2} }}\sqrt {1 + T_{1} T_{2} \Omega_{R}^{2} } .$$

It is worth noting here that Eq. (13) is exactly the same as that of ODMR if its laser pumping rate is set to 0^47^. It can be further shown that

14 $$C\left( \Omega \right) \equiv \frac{{T_{b} \left( {\Omega ,\delta = \infty } \right) - T_{b} \left( {\Omega ,\delta = 0} \right)}}{{T_{b} \left( {\Omega ,\delta = \infty } \right)}} = \frac{{T_{1} T_{2} \Omega_{R}^{2} }}{{1 + T_{1} T_{2} \Omega_{R}^{2} }}.$$

The term $C\left( \Omega \right)$ here can be regarded as the contrast of the optically detected relaxation spectrum at the center of the spin resonance. It has a logistic (or sigmoidal) form if Eq. (14) is written as a function of MW power ($P_{dbm}$) in decibel unit (See Supplementary Information for details). Figure 6a compares the numerically calculated result from Eq. (14) with the measurement performed at $\Omega_{R} {/}2\pi$ = 0.061 MHz, both consistently showing a smaller change in $T_{b}$ as $\left| \delta \right|$ increases. Moreover, the dependence of $T_{b}$ on $\omega$ (or $\delta$) is Lorentzian-like, which is also in agreement with the experimental finding. However, from Eq. (13) and using the experimentally measured values of $T_{1}$ = 1491 μs and $T_{2}$ = 1.7 μs, we obtained a linewidth of only 3.6 MHz, which is about threefold smaller than our experimental value of ~ 11 MHz.

The large discrepancies between experiments and simulations shown above led us to take into account the effect of inhomogeneous broadening on the spin transitions, which is a prominent feature in the ODMR spectra of NV^−^ spin ensembles in type-Ib diamond. The broadening results from random detuning ($\delta \ne$ 0) due to coupling between spins in the spin bath, which complicates observation as well as analysis. To examine the effect of inhomogeneous broadening, we first acquired the ODMR spectra at low MW powers to reveal the hyperfine structures of the NV^−^ ensembles. These hyperfine structures, which are linked to the intrinsic ^14^N nuclear spins in the hosting matrix, showed three overlapping bands with a total width of ~ 7 MHz, obtained by fitting with a single Lorentzian function. This hyperfine overlapping width $\Delta \omega_{fs}$ is the same as a previously reported value for a dense ensemble of NV^−^ centers in type-Ib diamond^47^. Taking the Lorentzian function as the detuning probability distribution, we convolute it with Eq. (11) by

15 $$T_{total} \left( {\Omega ,\delta } \right) = \frac{1}{\pi }\mathop \smallint \limits_{ - \infty }^{\infty } T_{b} \left( {\Omega ,x} \right)\frac{{\frac{{\Delta \omega_{fs} }}{2}}}{{\left( {\frac{{\Delta \omega_{fs} }}{2}} \right)^{2} + \left( {x - \delta } \right)^{2} }}dx,$$

and attain

16 $$T_{total} \left( {\Omega ,\delta } \right) = T_{1} \left[ {1 - \frac{{\left( {T_{1} - T_{2} } \right)\Omega_{R}^{2} }}{{T_{2} }} \cdot \frac{{\Delta \omega_{b} + \Delta \omega_{fs} }}{{\Delta \omega_{b} }} \cdot \frac{1}{{\left( {\frac{{\Delta \omega_{b} + \Delta \omega_{fs} }}{2}} \right)^{2} + \delta^{2} }}} \right].$$

The new spectrum is also Lorentzian-like and has a full width at half maximum of $\Delta \omega_{b} + \Delta \omega_{fs}$. In Fig. 6a, we also show the results of the experiment and the numerical simulation using Eq. (16) and $\Delta \omega_{fs} {/}2\pi$ = 7 MHz. The magnetic field and light power were the same as the condition for ODMR in Fig. 3b. A remarkably good agreement between measurements and simulation was achieved over the entire region of interest in the spectrum. Such MW frequency dependence hints for metrology applications of this method in a way analogous to the ODMR-based sensing protocols for magnetic field, temperature, etc.^3^. However, there is no light narrowing as observed by ODMR because of the lack of laser pumping during MW irradiation in this spin relaxation experiment^47^.

Many factors may contribute to the remaining discrepancies between experiment and simulation in Fig. 6b at the high MW power region. One of the possible factors is the coupling of the spin states with the MW driving fields, known as the dressed states^50–53^, at $\Omega_{R} {/}2\pi$ > 0.1 MHz. In these dressed states, the mechanism of the decoherence, the MW strength dependence, and the effect of off-resonance by inhomogeneous broadening vastly differ from the present treatment. Although the dressed state analysis is beyond the scope of this work, some useful information can be obtained from the recent studies by Golter et al.^50^ who found that the decoherence of NV^−^ spins in diamond could be suppressed by dressing the states with MW at a coupling rate near 1 MHz, leading to a 50-fold reduction in the spin transition linewidths. Morishita, et al.^53^ also demonstrated an extension of the $T_{2}$ coherence time for a single NV^−^ center in diamond at a Rabi frequency of ~ 1 MHz with a MW driving power of 33 µT and a MW driving frequency 2834.75 MHz. Our observation of the significantly longer coherence time than that predicted by the simulation based on the classical Bloch theory at $\Omega_{R} {/}2\pi$ > 0.1 MHz (cf. Fig. 6b) appears to be consistent with the dressed state model. It will also be of interest to investigate the spin bath interaction with open quantum system methods (e.g. hierarchy of stochastic pure states^54^), where signatures of non-Markovian behaviors might be identified. These interesting possibilities of research directions together with the use of well oriented diamond samples containing single NV^−^ centers are left for future exploration.

## Conclusion

First described by Torrey in 1949^35^, the baseline decay in damped Rabi oscillation is a fundamental spin property that has not yet been well explored. The NV^−^ spins in diamond provide an excellent opportunity for in-depth study of this property. Using dense NV^−^ ensembles, we have experimentally measured the baseline decays as functions of MW power and frequency. In line with the prediction based on the Bloch formalism for a simple two-level system, the baseline decay time constants were found to decrease with increasing MW field strength and show a Lorentzian-like spectrum with respect to MW detuning. Good agreement between observations and simulation can be reached only after properly taking into account the inhomogeneous band broadening effect in the low MW power region. Studies on such a high-density spin defect system offer new insight into their potential applications in quantum metrology. Moreover, they pave ways to better understanding of this spin property with single NV^−^ centers that should provide a more rigorous test of the theory. The experimental protocols and theoretical modeling developed in this work for NV^−^ ensembles are readily adaptable to other quantum systems as well.

## Method

The experimental setup consisted of a 532-nm CW laser (Coherent, Verdi OPSL) for optical pumping and probing, a 40 × objective lens (Nikon) with N.A. = 0.6 for focusing laser light and collecting fluorescence emission, and an ICCD camera (Andor, iStar DH712) for photon detection and fluorescence imaging. The laser light was fed through a double-pass optical path to an acoustic optical modulator (Isomet, 1250C) for light pulse control. To obtain the signals primarily from NV^−^ centers, a 665 nm long-pass filter was placed in front of the camera. For electron spin manipulation, a MW synthesizer (Windfreak Technologies, SynthHD pro v2) together with a MW amplifier (Mini-circuits, ZHL-16W-43S+) were employed. The MW power through the amplifier was linearly calibrated with a power meter. The sample consisted of a fluorescent microdiamond (FMD), which was illuminated with a defocused 532-nm laser at ~ 7 mW over an area of roughly 25 μm × 25 μm (~ 1 kW/cm^2^) throughout the entire work to avoid charge ionization but yet enable initialization of the spins within a reasonable time. The ensemble spin sublevels were excited by MW fed through a gold wire^55^ within a distance of 5 μm near the FMD of interest as observed on the wide-field image. In separate experiments, a defocused 532-nm laser beam was used to measure the NV charge recombination rates at different laser power settings (7 mW or 70 mW).

## Supplementary Information

Supplementary Information.
