# Supplementary material for: Relaxation of a dense ensemble of spins in diamond under a continuous microwave driving field
Source: Sci Rep. 2021 Aug 11;11:16278. doi: 10.1038/s41598-021-95722-z (PMC8358020; doi:10.1038/s41598-021-95722-z)
Supplement: Supplementary file 1 — Supplementary Information. [file 41598_2021_95722_MOESM1_ESM.pdf]

Supplementary Information

Charge dynamics

Figure S1 shows two photoluminescence time traces of the NV^- ensembles in FMD excited by a green laser with powers of 7 mW and 70 mW for optically induced spin polarization. Measured in the dark without MW, the photoluminescence intensities peaked at $t \approx 10$ μs and then decreased exponentially with time. We fitted the fluorescence decay data with an empirical coupled charge-spin formula as

$$I(t) = I_0 \left[1 - \alpha \cdot \exp\left(-\sqrt{\frac{t}{T_{rc}}} \right) + \beta \cdot \exp\left(-\sqrt{\frac{t}{T_{off}}} \right) \right], \tag{S1}$$

where α is the charge conversion amplitude, β is the spin amplitude, and T_{rc} is the recharging time constant. We obtained α = 0.053 and 0.104, β = 0.135 and 0.296, T_{rc} = 1.55 and 1.55 μs, and T_{off} = 1491 and 1491 μs at the laser power of 7 mW and 70 mW, respectively. The charge recombination effect clearly is more prominent at higher laser powers.

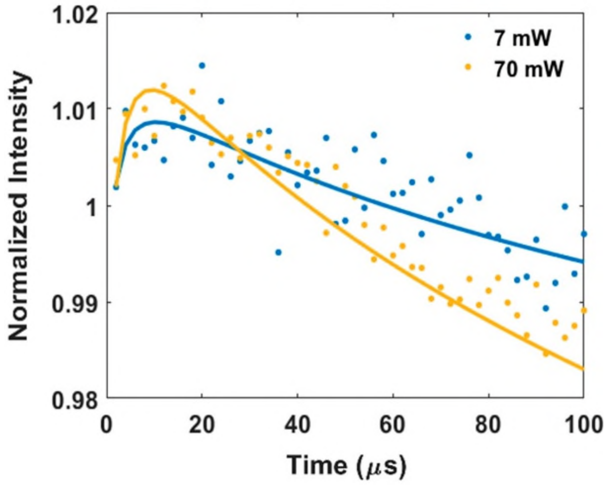

Fig. S1. Variation of the photoluminescence intensity over time during optically induced spin polarization of a dense ensemble of NV^- centers in FMD. The data acquired with a laser power of 7 mW or 70 mW were normalized with respect to the intensity at t = 0.

Double-stretch exponential decay

In a diamond crystal, the 4 orientations of NV centers could be categorized into 4 crystallographic groups. In the presence of a static magnetic field, these 4 groups of NV centers can be distinguished spectrally on ODMR spectrum as shown in **Figure 3(b)**. By applying a MW frequency resonant with one of the 8 Zeeman-split peaks on ODMR spectrum, we were able to independently control a specific group of the NV center ensemble. The decay process of the resonant peak will take on a MW-driven time constant T_b, while the other non-driven peaks behave like Eqn. (2) with the time constant T_{off}. The total contribution from every NV center has the form

$$I(t) = I_0 \left[A \cdot \exp\left(-\sqrt{\frac{t}{T_b}} \right) + B \cdot \exp\left(-\sqrt{\frac{t}{T_{off}}} \right) \right], \tag{S2}$$

where A is the contribution from the resonant peak and B is the contribution from all the other peaks combined.

Intuitively speaking, the 3 non-driven groups occupy 3/4 of the contribution to the total fluorescence intensity because the contribution ratio is proportional to the population ratio. The MW-driven group, however, is more complicated. Due to the difference in decay rate, the two transitions $|m_s = 0\rangle \rightarrow |m_s = -1\rangle$ and $|m_s = 0\rangle \rightarrow |m_s = +1\rangle$ of the driven group will have different equilibrium population. Let P_r be the equilibrium population ratio of the resonant sublevel in the driven group and P_{nr} be the equilibrium population ratio of non-resonant sublevel in the driven group. Since the driven group in sum occupies only 1/4 contribution to the total fluorescence intensity, we have $P_r + P_{nr}$ = 1/4. To obtain P_r and P_{nr}, a good approximation is that the equilibrium population ratio of the two transitions in the driven group is inversely proportional to their respective decay time, i.e., $P_r/P_{nr} = T_{off}/T_b$, from which we could deduce

$$P_r = \frac{T_{off}}{4(T_{off} + T_b)} \tag{S3}$$

and

$$P_{nr} = \frac{T_b}{4(T_{off} + T_b)}. \tag{S4}$$

By comparing the result with Eqn. (S2), we have $A = P_r$ and $B = P_{nr} + 3/4$. Plugging A and B back into Eqn. (S2) yields Eqn. (3) in the main text.

MW-driven spin relaxation

The general solution of Eqn. (6) is

$$\lambda = -\frac{2}{3}(\Gamma_1 + \Gamma_2) + \sqrt[3]{u1 - \sqrt{u2}} + \sqrt[3]{u1 + \sqrt{u2}}, \tag{S5}$$

where

$$u1 = -\frac{8}{27}(\Gamma_1 + \Gamma_2)^3 + \frac{1}{3}(\Gamma_1 + \Gamma_2)(4\Omega^2 + 4\Gamma_1\Gamma_2 + \Gamma_2^2 + \delta^2) - (2\Omega^2\Gamma_2 + \Gamma_1\Gamma_2^2 + \Gamma_1\delta^2), \tag{S6}$$

and

$$u2 = u1^2 + \left[-\frac{4}{9}(\Gamma_1 + \Gamma_2)^2 + \frac{1}{3}(4\Omega^2 + 4\Gamma_1\Gamma_2 + \Gamma_2^2 + \delta^2)\right]^3. \tag{S7}$$

To obtain the exact form of Q in Eqn. (11), we apply a Rabi frequency Ω such that

$$\Omega^2 = Q, \tag{S8}$$

from which we can derive

$$T_b(\sqrt{Q}) = \frac{1}{2}(T_1 + T_2) = \frac{1}{2}\left(\frac{1}{2\Gamma_1} + \frac{1}{\Gamma_2}\right). \tag{S9}$$

Substituting the above result into Eqn. (10), we have

$$\Omega^2 = \frac{\Gamma_1\left[4{\Gamma_1}^2({\Gamma_2}^2 + \delta^2) + {\Gamma_2}^2({\Gamma_2}^2 + \delta^2) - 4\Gamma_1({\Gamma_2}^3 - \Gamma_2\delta^2)\right]}{2\Gamma_2(2\Gamma_1 + \Gamma_2)^2} \tag{S10}$$

Comparing it with the formula (S8) yields

$$Q(\delta) = \frac{\Gamma_1\Gamma_2}{2}\left(\frac{\Gamma_2 - 2\Gamma_1}{\Gamma_2 + 2\Gamma_1}\right)^2 + \frac{\Gamma_1\delta^2}{2\Gamma_2}, \tag{S11}$$

which is reduced to Eqn. (12) for $\Gamma_2 \gg 2\Gamma_2$ or $T_1 \gg T_2$. The final form of Eqn. (11) under the condition $T_1 \gg T_2$ is

$$T_b(\Omega, \delta) = (T_1 - T_2)\left(1 - \frac{4T_1T_2\Omega^2}{1 + 4T_1T_2\Omega^2 + T_2^2\delta^2}\right) + T_2, \tag{S12}$$

Figure S2 compares the results calculated with Eqn. (S9) and the results calculated with Eqn. (6) for the real eigenvalues using T_1 = 1000 μs and T_2 = 1 μs at the MW detuning of $\delta/2\pi$ = 0, 0.5, and 1 MHz. Good agreement is reached at $\delta/2\pi$ > 0 MHz.

One may express the contrast $C(\Omega)$ in Eqn. (14) in terms of MW power (P) in dBm unit (P_{dbm}), which is a convenient measure of absolute power. It is known that P is linearly proportional to Ω^2 by

$$P \propto \Omega^2 = \Omega_0^2 10^{\frac{P_{dbm}}{10}} = \Omega_0^2 e^{\frac{ln(10)}{10}P_{dbm}}, \tag{S13}$$

where Ω_0 is a constant. We can then rewrite Eqn. (14) in a logistic (or sigmodal) form as

$$C(\Omega) = \frac{1}{1 + 1/(4T_1T_2\Omega^2)} = \frac{1}{1 + \exp(-kP_{dbm} + c)}, \tag{S14}$$

where k = ln (10)/10 = 0.230259 and $c \equiv -log(4T_1T_2\Omega_0^2)$.

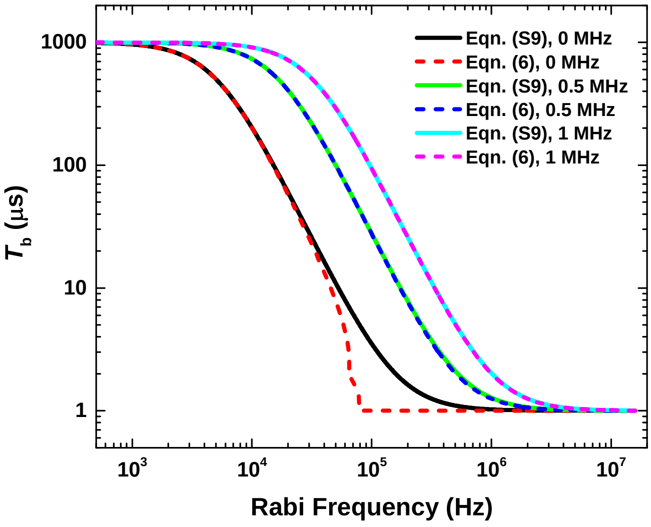

Fig. S2. Variation of MW-driven baseline decay times with MW powers expressed in terms of Rabi frequency at the detuning of $\delta/2\pi$ = 0, 0.5, and 1 MHz. Solid curves are calculated results with Eqn. (S9) and dashed curves are calculated results from Eqn. (6) for the real eigenvalues using T_1 = 1000 μs and T_2 = 1 μs.

Zero detuning

At $\delta = 0$, we have

$$(\lambda + \Gamma_2)[\lambda^2 + (2\Gamma_1 + \Gamma_2)\lambda + 2\Gamma_1\Gamma_2 + 4\Omega^2] = 0, \tag{S15}$$

and the three solutions of this characteristic polynomial are

$$\lambda_{1,2,3} = \frac{-(2\Gamma_1 + \Gamma_2) \pm \sqrt{(2\Gamma_1 - \Gamma_2)^2 - 16\Omega^2}}{2}, -\Gamma_2 \tag{S16}$$

No Rabi oscillation could be found if $(2\Gamma_1 - \Gamma_2)^2 - 16\Omega^2 \geq 0$ or $|1/T_1 - 1/T_2| \geq 2\Omega_R$, as all three solutions are real eigenvalues. This condition can be met at low MW powers (i.e. small Ω_R) for single NV⁻ center in a dilute spin bath, which have near zero random detuning from nearby environment. The third eigenvalue shows no Ω dependence and thus can be ignored.

Being a negative inverse of λ, the MW-driven baseline decay time constant $T_b(\Omega)$ has the form

$$T_b(\Omega) \equiv -\frac{1}{\lambda} = \frac{(2\Gamma_1 + \Gamma_2) \pm \sqrt{(2\Gamma_1 - \Gamma_2)^2 - 16\Omega^2}}{4\Gamma_1\Gamma_2 + 8\Omega^2}. \tag{S17}$$

Given the constrain that $T_b(\Omega) \leq T_1$ at $\Omega \geq 0$, we have

$$T_b(\Omega) = \frac{(T_1 + T_2) + \sqrt{(T_1 - T_2)^2 - 4T_1^2T_2^2\Omega_R^2}}{2(1 + T_1T_2\Omega_R^2)}. \tag{S18}$$

The other solution applies to $T_b(\Omega) \geq T_2$ at $\Omega \geq 0$.
